# Diabetes mellitus in patients with chronic obstructive pulmonary disease-The impact on mortality

**DOI:** 10.1371/journal.pone.0175794

**Published:** 2017-04-14

**Authors:** Te-Wei Ho, Chun-Ta Huang, Sheng-Yuan Ruan, Yi-Ju Tsai, Feipei Lai, Chong-Jen Yu

**Affiliations:** 1 Graduate Institute of Biomedical Electronics and Bioinformatics, National Taiwan University, Taipei, Taiwan; 2 Department of Internal Medicine, National Taiwan University Hospital, Taipei, Taiwan; 3 Department of Traumatology, National Taiwan University Hospital, Taipei, Taiwan; 4 Graduate Institute of Clinical Medicine, National Taiwan University, Taipei, Taiwan; 5 Graduate Institute of Biomedical and Pharmaceutical Science, College of Medicine, Fu Jen Catholic University, New Taipei City, Taiwan; National and Kapodistrian University of Athens, GREECE

## Abstract

**Background:**

Chronic obstructive pulmonary disease (COPD) is the leading cause of morbidity and mortality worldwide. There is evidence to support a connection between COPD and diabetes mellitus (DM), another common medical disorder. However, additional research is required to improve our knowledge of these relationships and their possible implications. In this study, we investigated the impact of DM on patient outcomes through the clinical course of COPD.

**Methods:**

We conducted a cohort study in patients from the Taiwan Longitudinal Health Insurance Database between 2000 and 2013. Patients with COPD were identified and assessed for pre-existing and incident DM. A Cox proportional hazards model was built to identify factors associated with incident DM and to explore the prognostic effects of DM on COPD patients. A propensity score method was used to match COPD patients with incident DM to controls without incident DM.

**Results:**

Pre-existing DM was present in 332 (16%) of 2015 COPD patients who had a significantly higher hazard ratio (HR) [1.244, 95% confidence interval (CI) 1.010–1.532] for mortality than that of the COPD patients without pre-existing DM. During the 10-year follow-up period, 304 (19%) of 1568 COPD patients developed incident DM; comorbid hypertension (HR, 1.810; 95% CI, 1.363–2.403), cerebrovascular disease (HR, 1.517; 95% CI, 1.146–2.008) and coronary artery disease (HR, 1.408; 95% CI 1.089–1.820) were significant factors associated with incident DM. Survival was worse for the COPD patients with incident DM than for the matched controls without incident DM (Log-rank, p = 0.027).

**Conclusions:**

DM, either pre-existing or incident, was associated with worse outcomes in COPD patients. Targeted surveillance and management of DM may be important in clinical care of the COPD population.

## Introduction

Chronic obstructive pulmonary disease (COPD) is characterized by persistent airflow limitation and is a global health issue with high social and economic costs.[1, 2] Development of COPD is associated with chronic bronchial and alveolar inflammation in response to noxious particles or gases, primarily those in tobacco smoking exposure.[2] In addition to these pulmonary abnormalities, COPD has a systemic component that includes significant extra-pulmonary effects, such as skeletal muscle dysfunction, weight loss, osteoporosis, and depression.[3] The pathogenetic mechanisms behind these systemic effects are poorly understood but are probably interrelated and multifactorial, including systemic inflammation, physical inactivity, tissue hypoxia, and oxidative stress, among others.[3] Extra-pulmonary manifestations of COPD may inversely lead to worsened dyspnea, impaired functional status, reduced health-related quality of life, and increased death risks of these patients.[4]

Diabetes mellitus (DM) is a major global metabolic disorder affecting approximately 300 million individuals worldwide.[5] Numerous studies have shown that low-grade chronic inflammation is part of the insulin resistance syndrome and is associated with development of DM.[6, 7] Accordingly, chronic systemic inflammation is probably one of the common denominators between COPD and DM. Epidemiological studies have found that DM is more frequent in COPD patients and likely to affect their prognosis.[8, 9] On the other hand, a series of studies have reported an association between DM and reduced lung function.[10–12] Undoubtedly, the relationships between these two complex diseases are complicated, and more research into this issue is required to foster our understanding of them.

Therefore, we investigated the epidemiology and prognostic role of pre-existing and incident DM and explored clinical factors associated with development of incident DM throughout the clinical course of COPD in a claims database cohort study.

## Methods

### Data source

This study employed a claims database cohort study design using data from the Longitudinal Health Insurance Database (LHID) from January 1, 2000 to December 31, 2013. Taiwan launched a single-payer National Health Insurance (NHI) program on March 1, 1995. As of 2014, 99.9% of Taiwan’s population was enrolled.[13] The LHID contained de-identified and encrypted medical claims made by one million NHI beneficiaries who were randomly selected from the entire NHI population. The LHID has been employed to study a variety of diseases, including COPD,[14–16] DM,[17–19] and many others. The access to and use of the LHID was approved by the National Health Research Institutes, which managed and constructed the database. The research ethics committee of the National Taiwan University Hospital waived the need for review board approval.

### Study sample

The study cohort consisted of all patients who had either received a diagnosis of COPD (International Classification of Diseases, 9th Revision, Clinical Modification [ICD-9-CM] codes 491, 492, and 496) during two or more outpatient visits within 12 months or were hospitalized with a primary diagnosis of COPD between January 1, 2001 and December 31, 2003. Cohort entry was marked by the date when patients received their first eligible diagnosis.

Subjects were excluded if they (1) had ICD-9-CM diagnosis codes for COPD in 2000, (2) were aged <40 years at the time of COPD diagnosis, (3) had a diagnosis of asthma (ICD-9-CM codes 493.xx) over the study period, (4) had <1-year follow-up after COPD diagnosis, (5) were diagnosed as having type 1 DM (ICD-9-CM codes 250.x1 and 250.x3) during the study period, and (6) had a prescription of COPD-related medications for <1 month within 1 year of COPD diagnosis. Patients with type 1 DM were excluded from this study because it had a well-known pathogenesis different from that of type 2 DM.[20]

### Data collection

The information about demographics, comorbidities, COPD medications, concomitant medications and COPD severity was retrieved from the LHID. Comorbidities of interest were selected on the basis of the findings of a previous study that assessed long-term outcomes in COPD[21] and included hypertension, dyslipidemia, cerebrovascular disease, heart failure, coronary artery disease, kidney disease, liver disease, and malignancy identified based on ICD-9-CM codes (S1 Table). COPD-related medications were defined as prescriptions containing corticosteroids, methylxanthines, anticholinergics, and β_2_-agonists. Concomitant medications obtained were those associated with symptomatology and outcomes of COPD, and included angiotensin-converting enzyme inhibitors (ACEIs),[22, 23] angiotensin II receptor blockers (ARBs),[22, 24] β blockers,[25, 26] calcium channel blockers,[27, 28] and statins.[14, 29] Since spirometry data were not available for the LHID, proxy indicators of COPD severity, including the number of COPD-related emergency services and COPD-related hospitalizations, were measured during the year following COPD diagnosis.

### Study endpoints and follow-up

The primary endpoint of the entire COPD cohort was all-cause mortality. For COPD patients without pre-existing DM, the primary endpoint also included development of incident DM. The diagnosis of DM was established by any hospitalization for DM or at least three outpatient diagnoses of DM within 1 year based on the ICD-9-CM codes 250.xx (except 250.x1 and 250.x3).[30] Pre-existing DM was defined as the presence of DM prior to or concomitantly with the diagnosis of COPD. When DM diagnosis was made after the date of cohort entry, it was referred to as incident DM. All patients were followed up until death, the end of December 2013, or discontinuation of enrollment in the NHI program, whichever came first.

### Statistical analyses

Categorical variables were represented by a number (%) and analyzed by the chi-square or Fisher’s exact test, as appropriate. Continuous variables were summarized as the mean ± standard deviation and compared by using Student’s t-test. To identify independent factors associated with all-cause mortality and development of incident DM, we constructed multivariate Cox proportional hazards models and reported hazard ratios (HRs) with 95% confidence intervals (95% CIs). Those variables with a p value of <0.05 in the univariate analyses were entered into the multivariate models. Time-to-event curves were generated by using the Kaplan–Meier method and compared by performing the log-rank test.

Since there were significant differences in the baseline characteristics between COPD patients with and without incident DM, propensity score matching was applied to balance potentially confounding factors when comparing outcome measures between the two groups.[31] In this study, the propensity score was the conditional probability of developing incident DM, as a binary dependent variable, under a set of measurements. We performed all matching on a one-to-one basis without replacement, and the caliper width was set to 0.25 times the standard deviation of the propensity score. The matching procedures were performed by using the Stata software, version 11 (StataCorp, College Station, TX).

Microsoft SQL Server 2008 was used for data management and computing. Data analysis was performed by using SPSS software (Version 22.0, 2012; SPSS Inc., Chicago, IL). A 2-tailed p value of <0.05 indicated statistical significance.

## Results

### Study population

**Fig 1:**
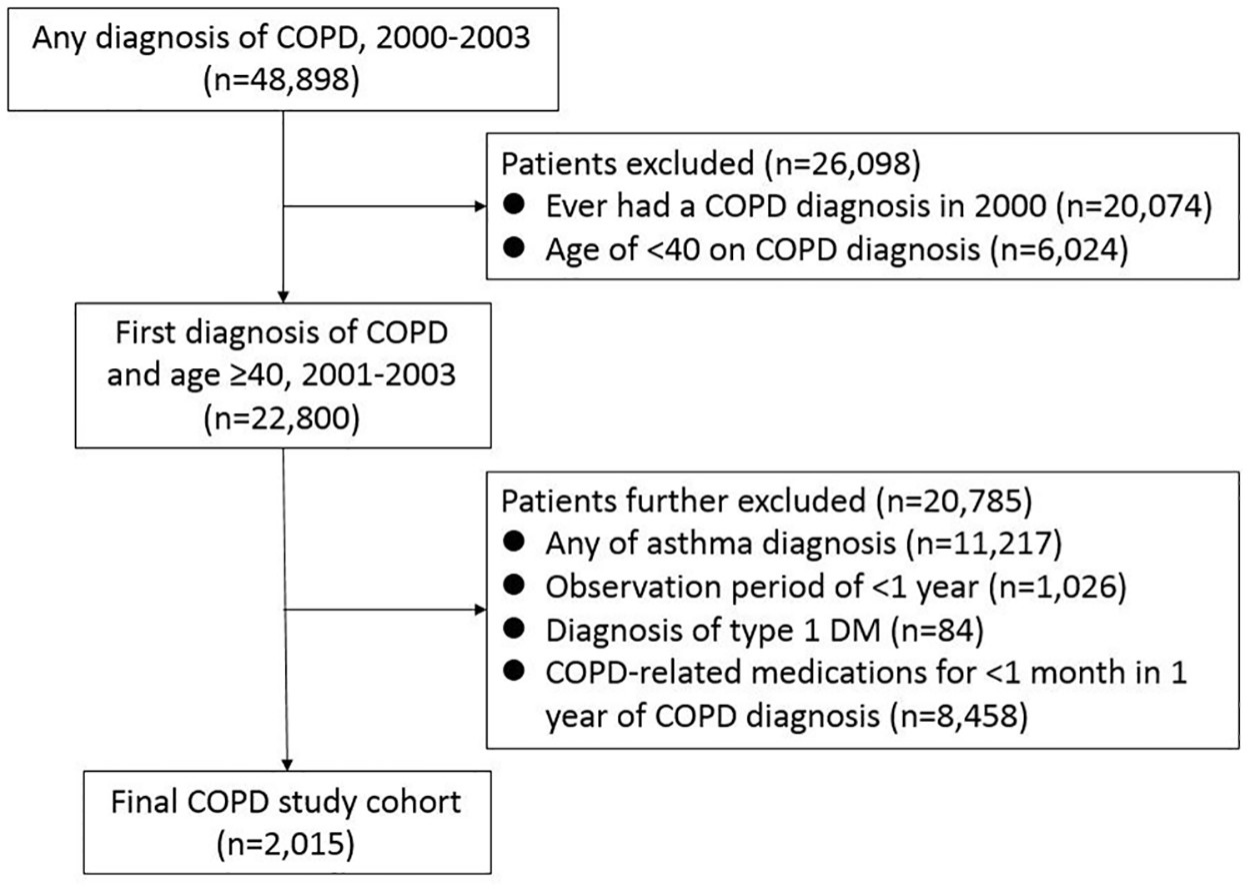
Study flow diagram. COPD, chronic obstructive pulmonary disease; DM, diabetes mellitus.

Over the study period, a total of 2,015 individuals were included as the study cohort (Fig 1). The mean age of the study population was 67.2 ± 12.0 years, and approximately 70% were male. Hypertension (50%) was the most common comorbidity, followed by coronary artery disease (23%) and cerebrovascular disease (18%).

### Pre-existing DM and its prognostic role

Pre-existing DM was identified in 332 (16%) patients on COPD diagnosis (Table 1). COPD patients with pre-existing DM had a higher proportion of comorbidities, including hypertension, coronary artery disease, cerebrovascular disease, heart failure, and dyslipidemia, than did those without pre-existing DM. ACEIs/ARBs, calcium channel blockers, and statins were more frequently prescribed in COPD patients with pre-existing DM than in those without.

**Table 1:** Characteristics of all study patients with and without pre-existing DM at COPD diagnosis (n = 2015).

Characteristics	DM (n = 332)	Non-DM (n = 1683)	p value
Age, years			
<50	20(6.0)	201(12)	0.008
50–59	65(20)	277(17)	
60–69	94(28)	415(25)	
≥70	153(46)	790(47)	
Male gender	204(61)	1214(72)	<0.001
Comorbidity			
Hypertension	211(64)	797(47)	<0.001
Dyslipidemia	35(11)	69(4.1)	<0.001
Cerebrovascular disease	81(24)	282(17)	0.001
Heart failure	52(16)	182(11)	0.015
Coronary artery disease	95(29)	378(23)	0.019
Kidney disease	31(9.3)	105(6.2)	0.054
Liver disease	31(9.3)	111(6.6)	0.079
ZMalignancy	33(9.9)	146(8.7)	0.460
Concomitant medications			
ACEI/ARB	40.1(40)	417(25)	<0.001
β blocker	28.6(29)	486(29)	0.947
Calcium channel blocker	47.0(47)	571(34)	<0.001
Statin	34(10)	43(2.6)	<0.001
COPD severity			
No ES or hospitalization	295 (89)	1525 (91)	0.256^a^
1 ES	23 (6.9)	106 (6.3)	
≥2 ES or hospitalization	14 (4.2)	52 (3.1)	

^a^ p for trend

ACEI, angiotensin-converting enzyme inhibitor; ARB, angiotensin II receptor blocker; COPD, chronic obstructive pulmonary disease; DM, diabetes mellitus; ES, emergency service

**Fig 2:**
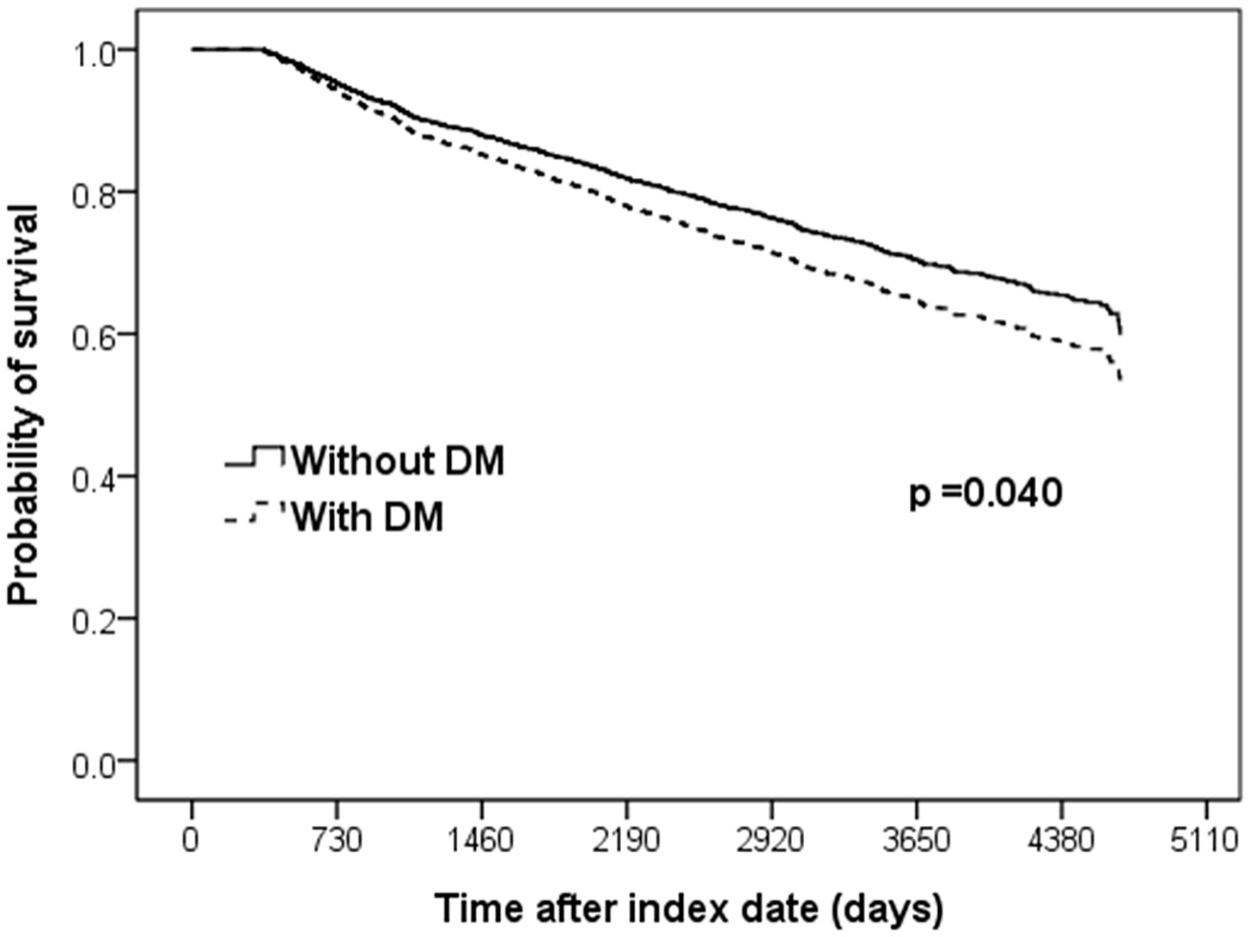
Adjusted Kaplan–Meier curves for survival in all COPD patients with and without pre-existing DM (n = 2015). ^a^Adjusted for age, gender, comorbidities (hypertension, dyslipidemia, cerebrovascular disease, heart failure, and coronary artery disease) and concomitant medications (ACEI/ARB, calcium channel blocker, and statin). ACEI, angiotensin-converting enzyme inhibitor; ARB, angiotensin II receptor blocker; COPD, chronic obstructive pulmonary disease; DM, diabetes mellitus.

Throughout the study period, a total of 670 (33%) COPD patients died, and the adjusted Kaplan–Meier curves (Fig 2) showed a higher probability of mortality in COPD patients with pre-existing DM than in those without pre-existing DM (Cox, p = 0.040). Multivariate Cox proportional hazards analysis (Table 2) indicated that age (HR, 1.859; 95% CI, 1.233–2.803 for age 60–69; HR, 4.905; 95% CI, 3.347–7.188 for age ≥70 years; with reference to age <50 years) and comorbidities, i.e., cerebrovascular disease (HR, 1.360; 95% CI, 1.125–1.644) and heart failure (HR, 1.823; 95% CI, 1.478–2.249), were also significant factors associated with mortality in the total COPD population. Given the associations between DM and comorbidities, such as hypertension, dyslipidemia, cerebrovascular disease, heart failure, and coronary artery disease, we checked their interactions with DM by using the likelihood ratio test and found no additive effects on patient mortality (p = 0.72, 0.91, 0.30, 0.70 and 0.75, respectively).

**Table 2:** Multivariate Cox proportional hazards model for mortality in all COPD patients (n = 2015).

Variables	Hazard ratio	95% confidence interval	p value
Pre-existing DM	1.244	1.010–1.532	0.040
Age, years			
<50	Reference		
50–59	1.416	0.912–2.198	0.121
60–69	1.859	1.233–2.803	0.003
≥70	4.905	3.347–7.188	<0.001
Male gender	1.588	1.316–1.916	<0.001
Comorbidity			
Hypertension	1.007	0.840–1.208	0.937
Dyslipidemia	0.639	0.405–1.010	0.055
Cerebrovascular disease	1.360	1.125–1.644	0.001
Heart failure	1.823	1.478–2.249	<0.001
Coronary artery disease	1.078	0.903–1.286	0.406
Concomitant medications			
ACEI/ARB	0.855	0.709–1.032	0.103
Calcium channel blocker	1.166	0.974–1.396	0.095
Statin	0.659	0.407–1.066	0.089

ACEI, angiotensin-converting enzyme inhibitor; ARB, angiotensin II receptor blocker; COPD, chronic obstructive pulmonary disease; DM, diabetes mellitus

### Features associated with development of incident DM

**Fig 3:**
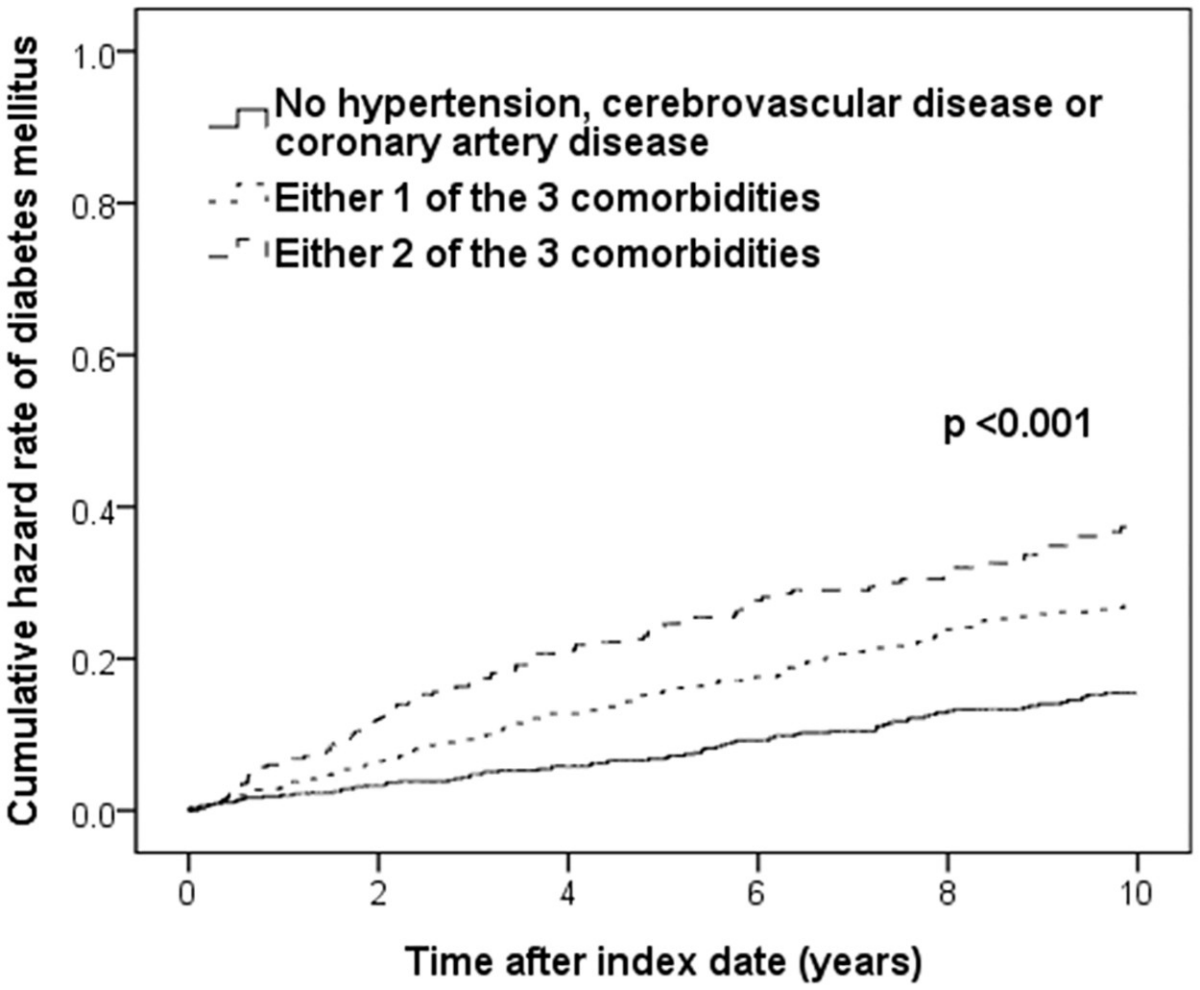
Cumulative hazard rate of incident DM in COPD patients according to comorbidity status (n = 1568). COPD, chronic obstructive pulmonary disease; DM, diabetes mellitus.

During the 10-year follow-up, 304 (19%) of the 1,568 COPD patients developed incident DM. The baseline features of the patients with and without incident DM are shown in Table 3. The prevalence of comorbidities and medication use were different between the two patient groups. Multivariate Cox proportional hazards analysis showed that comorbidities, including hypertension (HR, 1.810; 95% CI, 1.363–2.403), cerebrovascular disease (HR, 1.517; 95% CI, 1.146–2.008), and coronary artery disease (HR, 1.408; 95% CI, 1.089–1.820), were independent clinical factors associated with development of incident DM (Table 4). Compared with the COPD patients without these comorbidities (Fig 3), the patients with either one or two of the comorbidities were more likely to suffer from incident DM (Log-rank, p < 0.001).

**Table 3:** Comparison of features between COPD patients with and without incident DM (n = 1568).

Characteristics	COPD patients with incident DM	COPD patients without incident DM	p value
	n = 304	n = 1264	
Age, years			
<50	32(11)	159(13)	0.087^a^
50–59	54(18)	211(17)	
Z60-69	89(29)	290(23)	
≥70	129(42)	604(48)	
Male gender	222(73)	919(73)	0.943
Comorbidity			
Hypertension	187(62)	548(43)	<0.001
Dyslipidemia	15(4.9)	44(3.5)	0.240
Cerebrovascular disease	66(22)	199(16)	0.017
Heart failure	40(13)	125(9.9)	0.097
Coronary artery disease	90(30)	256(20)	0.001
Kidney disease	24(7.9)	76(6.0)	0.239
Liver disease	21(6.9)	81(6.4)	0.795
Malignancy	20(6.6)	116(9.2)	0.173
Concomitant medications			
ACEI/ARB	90(30)	291(23)	0.021
β blocker	110(36)	333(26)	0.001
Calcium channel blocker	129(42)	393(31)	<0.001
Statin	11(3.6)	24(1.9)	0.082
COPD medications			
β_2_-agonist, short-acting	87(29)	385(31)	0.578
β_2_-agonist, long-acting	45(15)	232(18)	0.155
Anticholinergic, short-acting	30(9.9)	171(14)	0.104
Methylxanthine	254(84)	1058(84)	0.931
Inhaled corticosteroid	11(3.6)	62(4.9)	0.448
Oral corticosteroid	72(24)	254(20)	0.181
COPD severity			
No ES or hospitalization	268(88)	1152(91)	0.277^a^
1 ES	22(7.2)	77(6.1)	
≥2 ES or hospitalization	13(4.3)	35(2.8)	

^a^ p for trend

ACEI, angiotensin-converting enzyme inhibitor; ARB, angiotensin II receptor blocker; COPD, chronic obstructive pulmonary disease; DM, diabetes mellitus; ES, emergency service

**Table 4:** Multivariate Cox proportional hazards model to identify clinical factors associated with incident DM among COPD patients (n = 1568).

Variables	Hazard ratio	95% confidence interval	p value
Comorbidity			
Hypertension	1.810	1.363–2.403	<0.001
Cerebrovascular disease	1.517	1.146–2.008	0.004
Coronary artery disease	1.408	1.089–1.820	0.009
Concomitant medications			
ACEI/ARB	0.903	0.688–1.187	0.465
β blocker	1.079	0.833–1.397	0.566
Calcium channel blocker	1.066	0.815–1.396	0.640

ACEI, angiotensin-converting enzyme inhibitor; ARB, angiotensin II receptor blocker; COPD, chronic obstructive pulmonary disease; DM, diabetes mellitus

### Prognostic role of incident DM

**Fig 4:**
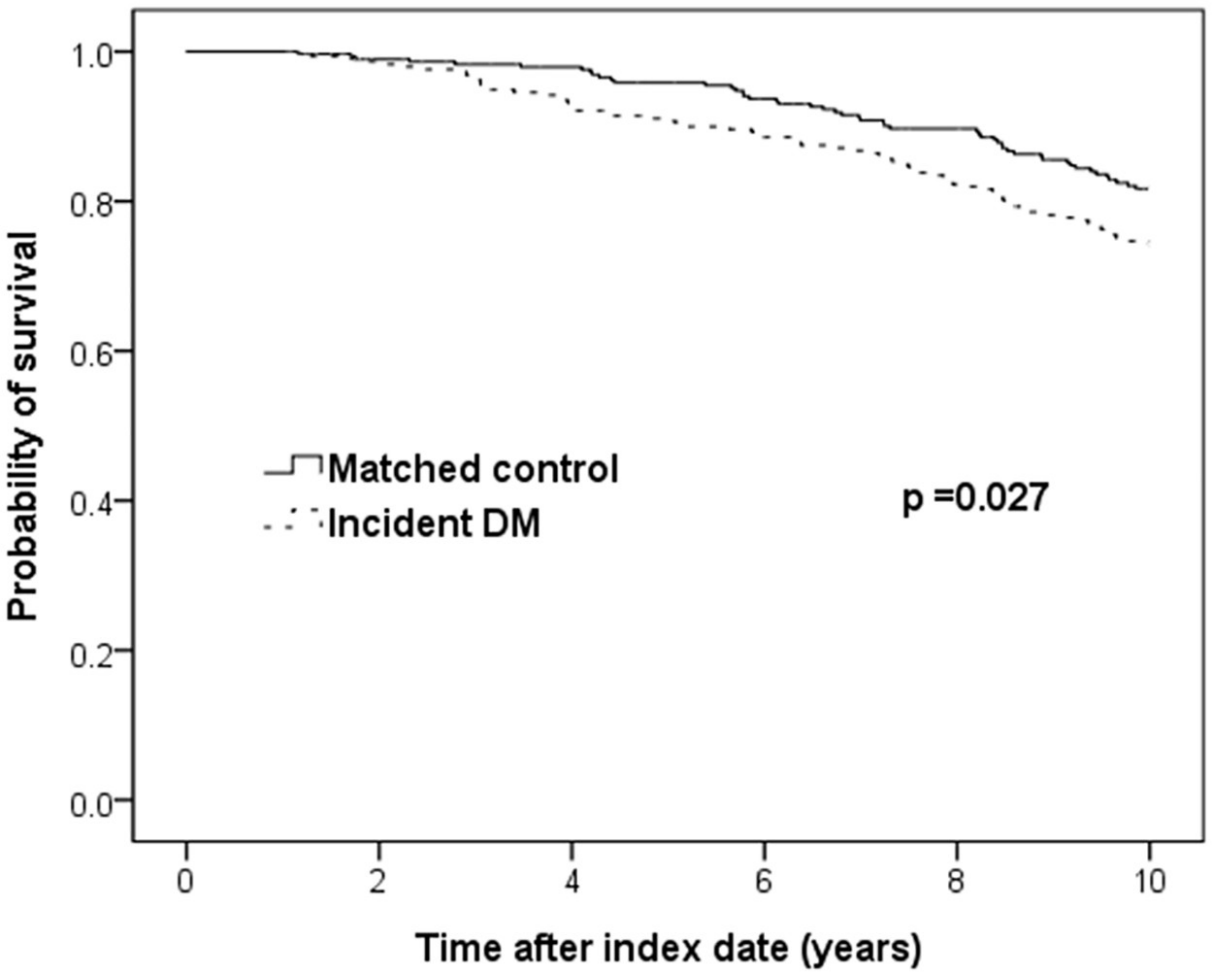
Kaplan-Meier curves for survival in COPD patients with incident DM and matched controls. COPD, chronic obstructive pulmonary disease; DM, diabetes mellitus.

After one-to-one matching for baseline characteristics, 304 COPD patients with incident DM were matched to those without incident DM. Table 5 presents the clinical features of patients in the propensity score-matched cohort. The age and gender distribution, comorbidity burden, use of concomitant and COPD medications, and COPD severity were similar between the two patient groups. The survival curves for the COPD patients with incident DM and their matched controls without incident DM are shown in Fig 4. In the analysis, survival was worse for the COPD patients with incident DM than for those without (Log-rank, p = 0.027).

**Table 5:** Comparison of features between COPD patients with incident DM and matched controls.

Characteristics	COPD patients with incident DM	Matched COPD controls	p value
	n = 304	n = 304	
Age, years	66.5±11.1	66.3±12.1	0.893
<50	32(11)	36(12)	0.880
50–59	54(18)	54(18)	
60–69	89(29)	81(27)	
≥70	129(42)	133(44)	
Male gender	222(73)	233(77)	0.304
Comorbidity			
Hypertension	187(62)	192(63)	0.676
Dyslipidemia	15(4.9)	16(5.3)	0.854
Cerebrovascular disease	66(22)	61(20)	0.618
Heart failure	40(13)	31(10)	0.256
Coronary artery disease	90(30)	81(27)	0.417
Kidney disease	24(7.9)	24(7.9)	0.999
Liver disease	21(6.9)	19(6.2)	0.744
Malignancy	20(6.6)	14(4.6)	0.290
Concomitant medications			
ACEI/ARB	90(30)	96(32)	0.597
β blocker	110(36)	112(37)	0.866
Calcium channel blocker	129(42)	133(44)	0.743
Statin	11(3.6)	9(3.0)	0.649
COPD medications			
β_2_-agonist, short-acting	87(29)	92(3)	0.656
β_2_-agonist, long-acting	45(15)	51(17)	0.505
Anticholinergic, short-acting	30(9.9)	40(13)	0.204
Methylxanthine	254(84)	256(84)	0.825
Inhaled corticosteroid	11(3.6)	16(5.3)	0.325
Oral corticosteroid	72(24)	57(19)	0.137
COPD severity			
No ES or hospitalization	268(88)	281(92)	0.244^a^
1 ES	22(7.2)	15(4.9)	
≥2 ES or hospitalization	13(4.3)	8(2.6)	

^a^ p for trend

ACEI, angiotensin-converting enzyme inhibitor; ARB, angiotensin II receptor blocker; COPD, chronic obstructive pulmonary disease; DM, diabetes mellitus; ES, emergency service

## Discussion

In this large claims database cohort study with a long follow-up period, both pre-existing and incident DM were associated with an increased risk of all-cause mortality in COPD patients. At the time of COPD diagnosis, 16% of the patients presented with pre-existing DM; moreover, 19% of those without pre-existing DM developed incident DM within 10 years of diagnosis of COPD. The presence of comorbidities, including hypertension, cerebrovascular disease, and coronary artery disease, were significantly associated with development of incident DM. In short, DM, either pre-existing or incident, was associated with worse outcomes for COPD patients through their clinical course.

In line with a recent multicenter investigation,[32] we found that pre-existing DM co-existing with COPD increased the hazards of mortality. Comorbid DM has also been shown to be associated with an increase in the length of hospital stay, in-hospital mortality, and long-term mortality following acute exacerbations of COPD.[33–35] The mechanisms responsible for the deleterious effects of DM on COPD prognosis are not fully understood and are likely to be multifactorial. For instance, impaired lung function has been observed in DM patients and may be the result of direct exposure to hyperglycemia.[36, 37] A rat study has demonstrated that the impact of hyperglycemia on the respiratory system is characterized by increased oxidative stress, structural changes in the lung tissue, and altered gas exchange.[38] Further, several pieces of clinical evidence indicate that hyperglycemia can drive inflammatory responses,[32, 39] which could subsequently lead to reduced pulmonary function and restrictive abnormalities in the lung tissue. Thus, reduced lung volumes and airflow limitation might be regarded as chronic complications of DM[12] and lead to adverse effects in patients suffering from COPD. On the other hand, DM would render COPD patients vulnerable to bacterial lung infection. Hyperglycemia can directly promote or sustain bacterial growth in the airways,[40] and impaired phagocytic function of polymorphonuclear leukocytes has been seen in DM patients.[41] Therefore, susceptibility to bacterial infection may contribute to worse outcomes in COPD patients with DM.

Recent evidence also supports that COPD constitutes an important risk factor for developing incident DM.[42, 43] Specifically, our study identified clinical features, i.e., hypertension, cerebrovascular disease, and coronary artery disease, that were associated with a higher probability of DM development in COPD patients, and these comorbidities in combination exerted additive effects on the incidence of DM. This may not be an unexpected finding. It has been observed that patients with COPD are more likely to suffer from metabolic syndrome and cardiovascular disease,[42, 44] and the pathophysiology predisposing to these disorders, such as physical inactivity, systemic inflammation, and tobacco smoking,[42, 44, 45] is almost the same as that leading to development of DM. Thus, COPD patients, in particular with comorbid hypertension, cerebrovascular disease, and coronary artery disease, are prone to experience incident DM. Further, recent reviews have concluded that COPD alone can be considered to be a risk factor for development of DM, as supported by several nationwide and international epidemiological studies.[46, 47] More importantly, in this study, we demonstrated that incident DM, similar to pre-existing DM, had a significant prognostic impact on patients with COPD. Taken together, although routine screening of patients with COPD for the presence of incident DM is not recommended in the current COPD practice guideline,[2] the findings herein and from other studies suggest that survey of dysglycemia in COPD patients should be performed, particularly when the patients carry certain warning comorbidities.

Comorbidities are commonly encountered in COPD patients of any severity and significantly affect their health status and prognosis.[48, 49] Cardiovascular disease is a major and important comorbidity in COPD.[50] Consistent with previous studies,[51, 52] we showed that comorbid heart failure appeared to significantly affect survival of COPD patients. Moreover, COPD and heart failure may be confused because they share common symptoms of breathlessness, cough, and wheezing.[53] Therefore, caution is needed for the diagnosis and management of heart failure in the COPD population.

Cerebrovascular disease is another comorbidity described as being more prevalent in patients with COPD,[9] probably through sharing common risk factors of aging, smoking, and genetic predisposition with COPD or its risk factors of physical inactivity, vasculopathy, and disturbed oxygenation that are aggravated by COPD.[54] Despite the clinical relevance of cerebrovascular disease in patients with COPD, little if any literature regarding its impact on COPD is available. The present study showed an adverse effect of comorbid cerebrovascular disease on prognosis of COPD patients. The underlying mechanisms are uncertain; however, it has been reported that patients with cerebrovascular disease have impaired lung function due to respiratory muscle weakness or central diaphragmatic impairment,[55] and these patients are also at increased risk of dysphagia and aspiration pneumonia.[56] All of these factors may have a role in modulating the clinical course of COPD patients and, thus, negatively affect their outcomes.

Our study had several potential limitations. First, information, such as body weight, smoking status, and spirometry data, was not available in the LHID. However, we used dyslipidemia, hypertension, and medical service utilization for COPD as proxy measures for these variables, so they may not markedly confound our study results. Second, only the diagnosis of DM has been validated in the LHID;[30] therefore, the accuracy of COPD diagnosis is unknown. However, the ICD-9-CM codes used for the diagnosis of COPD have been extensively used in other studies,[14, 57, 58] and the prescriptions of COPD-related medications and the age limit of ≥40 years were the prerequisite components for COPD diagnosis in the present study. Moreover, the baseline prevalence of pre-existing DM in our study (16%) was similar to that in other telephone interview surveys and prospective studies (12%–13%) in Taiwan.[59, 60] These strengths support the validity of our COPD diagnosis and the comparability of our study sample to the target population. Third, a substantial variation in the pharmacologic therapy of COPD patients between Taiwan and American–European countries,[61, 62] such as fewer prescriptions of inhaled corticosteroids and far more frequent use of theophyllines in Taiwan, could hinder the generalizability of our study results. Yet, the cultural, ethnic, or loco-regional differences in the clinical management of COPD also highlight the importance of our study and indicate that the detrimental effects of DM in patients with COPD go beyond social and environmental boundaries.

In conclusion, DM is a prevalent comorbidity at the time of COPD diagnosis, and it is not uncommon for COPD patients, particularly those with comorbid hypertension, cerebrovascular disease, and coronary artery disease, to develop DM during their disease course. Moreover, we demonstrated that both pre-existing and incident DM had a poor prognostic effect on COPD survival. Therefore, this study suggests that targeted surveillance and management of DM are important in clinical care of the COPD population.

## Supporting information

S1 Table Definitions of comorbidities. (DOCX)

## References

[1] Global Burden of Disease Study C. Global, regional, and national incidence, prevalence, and years lived with disability for 301 acute and chronic diseases and injuries in 188 countries, 1990–2013: a systematic analysis for the Global Burden of Disease Study 2013. Lancet. 2015;386(9995):743–800. 10.1016/S0140-6736(15)60692-4 26063472 PMC4561509

[2] Global Initiative for Chronic Obstructive Lung Disease. Global Strategy for the Diagnosis, Management and Prevention of COPD, Global Initiative for Chronic Obstructive Lung Disease (GOLD) 2017 http://goldcopd.org.

[3] Agusti AG. Systemic effects of chronic obstructive pulmonary disease. Proc Am Thorac Soc. 2005;2(4):367–70; discussion 71–2. 10.1513/pats.200504-026SR 16267364

[4] Barnes PJ, Celli BR. Systemic manifestations and comorbidities of COPD. Eur Respir J. 2009;33(5):1165–85. 10.1183/09031936.00128008 19407051

[5] Danaei G, Finucane MM, Lu Y, Singh GM, Cowan MJ, Paciorek CJ, et al National, regional, and global trends in fasting plasma glucose and diabetes prevalence since 1980: systematic analysis of health examination surveys and epidemiological studies with 370 country-years and 2.7 million participants. Lancet. 2011;378(9785):31–40. 10.1016/S0140-6736(11)60679-X 21705069

[6] Crook M. Type 2 diabetes mellitus: a disease of the innate immune system? An update. Diabet Med. 2004;21(3):203–7. 15008827 10.1046/j.1464-5491.2003.01030.x

[7] Festa A, D'Agostino R Jr., Howard G, Mykkanen L, Tracy RP, Haffner SM. Chronic subclinical inflammation as part of the insulin resistance syndrome: the Insulin Resistance Atherosclerosis Study (IRAS). Circulation. 2000;102(1):42–7. 10880413 10.1161/01.cir.102.1.42

[8] Mannino DM, Thorn D, Swensen A, Holguin F. Prevalence and outcomes of diabetes, hypertension and cardiovascular disease in COPD. Eur Respir J. 2008;32(4):962–9. 10.1183/09031936.00012408 18579551

[9] Feary JR, Rodrigues LC, Smith CJ, Hubbard RB, Gibson JE. Prevalence of major comorbidities in subjects with COPD and incidence of myocardial infarction and stroke: a comprehensive analysis using data from primary care. Thorax. 2010;65(11):956–62. 10.1136/thx.2009.128082 20871122

[10] Cazzola M, Calzetta L, Rogliani P, Lauro D, Novelli L, Page CP, et al High glucose enhances responsiveness of human airways smooth muscle via the Rho/ROCK pathway. Am J Respir Cell Mol Biol. 2012;47(4):509–16. 10.1165/rcmb.2011-0449OC 22652200

[11] Ehrlich SF, Quesenberry CP Jr., Van Den Eeden SK, Shan J, Ferrara A. Patients diagnosed with diabetes are at increased risk for asthma, chronic obstructive pulmonary disease, pulmonary fibrosis, and pneumonia but not lung cancer. Diabetes Care. 2010;33(1):55–60. 10.2337/dc09-0880 19808918 PMC2797986

[12] Davis WA, Knuiman M, Kendall P, Grange V, Davis TM, Fremantle Diabetes S. Glycemic exposure is associated with reduced pulmonary function in type 2 diabetes: the Fremantle Diabetes Study. Diabetes Care. 2004;27(3):752–7. 14988297 10.2337/diacare.27.3.752

[13] National Health Insurance Administration, Ministry of Health and Welfare, Taiwan, R.O.C. (2014). National Health Insurance Annual Report 2014–2015.

[14] Wang MT, Lo YW, Tsai CL, Chang LC, Malone DC, Chu CL, et al Statin use and risk of COPD exacerbation requiring hospitalization. Am J Med. 2013;126(7):598–606 e2. 10.1016/j.amjmed.2013.01.036 23684060

[15] Ho TW, Tsai YJ, Ruan SY, Huang CT, Lai F, Yu CJ, et al In-hospital and one-year mortality and their predictors in patients hospitalized for first-ever chronic obstructive pulmonary disease exacerbations: a nationwide population-based study. PLoS One. 2014;9(12):e114866 10.1371/journal.pone.0114866 25490399 PMC4260959

[16] Lee CT, Mao IC, Lin CH, Lin SH, Hsieh MC. Chronic obstructive pulmonary disease: a risk factor for type 2 diabetes: a nationwide population-based study. Eur J Clin Invest. 2013;43(11):1113–9. 10.1111/eci.12147 24028296

[17] Wu CS, Gau SS, Lai MS. Long-term antidepressant use and the risk of type 2 diabetes mellitus: a population-based, nested case-control study in Taiwan. J Clin Psychiatry. 2014;75(1):31–8; quiz 8. 10.4088/JCP.13m08421 24502860

[18] Chen CH, Hsu CM, Lin CL, Chou AK, Jeng LB. The Development of Diabetes after Subtotal Gastrectomy with Billroth II Anastomosis for Peptic Ulcer Disease. PLoS One. 2016;11(11):e0167321 10.1371/journal.pone.0167321 27893867 PMC5125684

[19] Lin JC, Shau WY, Lai MS. Sex- and age-specific prevalence and incidence rates of sight-threatening diabetic retinopathy in Taiwan. JAMA Ophthalmol. 2014;132(8):922–8. 10.1001/jamaophthalmol.2014.859 24809869

[20] Leslie RD, Palmer J, Schloot NC, Lernmark A. Diabetes at the crossroads: relevance of disease classification to pathophysiology and treatment. Diabetologia. 2016;59(1):13–20. 10.1007/s00125-015-3789-z 26498592

[21] Almagro P, Salvado M, Garcia-Vidal C, Rodriguez-Carballeira M, Delgado M, Barreiro B, et al Recent improvement in long-term survival after a COPD hospitalisation. Thorax. 2010;65(4):298–302. 10.1136/thx.2009.124818 20388752

[22] Mancini GB, Etminan M, Zhang B, Levesque LE, FitzGerald JM, Brophy JM. Reduction of morbidity and mortality by statins, angiotensin-converting enzyme inhibitors, and angiotensin receptor blockers in patients with chronic obstructive pulmonary disease. J Am Coll Cardiol. 2006;47(12):2554–60. 10.1016/j.jacc.2006.04.039 16781387

[23] Mortensen EM, Copeland LA, Pugh MJ, Restrepo MI, de Molina RM, Nakashima B, et al Impact of statins and ACE inhibitors on mortality after COPD exacerbations. Respir Res. 2009;10:45 10.1186/1465-9921-10-45 19493329 PMC2697974

[24] Andreas S, Herrmann-Lingen C, Raupach T, Luthje L, Fabricius JA, Hruska N, et al Angiotensin II blockers in obstructive pulmonary disease: a randomised controlled trial. Eur Respir J. 2006;27(5):972–9. 10.1183/09031936.06.00098105 16446313

[25] Short PM, Lipworth SI, Elder DH, Schembri S, Lipworth BJ. Effect of beta blockers in treatment of chronic obstructive pulmonary disease: a retrospective cohort study. BMJ. 2011;342:d2549 10.1136/bmj.d2549 21558357 PMC3091487

[26] Angeloni E, Melina G, Roscitano A, Refice S, Capuano F, Lechiancole A, et al beta-Blockers improve survival of patients with chronic obstructive pulmonary disease after coronary artery bypass grafting. Ann Thorac Surg. 2013;95(2):525–31. 10.1016/j.athoracsur.2012.07.080 23040827

[27] Burghuber OC. Nifedipine attenuates acute hypoxic pulmonary vasoconstriction in patients with chronic obstructive pulmonary disease. Respiration. 1987;52(2):86–93. 3671896 10.1159/000195309

[28] Mols P, Naeije R, Hallemans R, Melot C, Lejeune P, Englert M. Central and regional hemodynamic effects of nitrendipine in normotensive patients with chronic obstructive lung disease. J Cardiovasc Pharmacol. 1986;8(1):77–81. 2419698 10.1097/00005344-198601000-00013

[29] Horita N, Miyazawa N, Kojima R, Inoue M, Ishigatsubo Y, Ueda A, et al Statins reduce all-cause mortality in chronic obstructive pulmonary disease: a systematic review and meta-analysis of observational studies. Respir Res. 2014;15:80 10.1186/1465-9921-15-80 25029928 PMC4118277

[30] Lin CC, Lai MS, Syu CY, Chang SC, Tseng FY. Accuracy of diabetes diagnosis in health insurance claims data in Taiwan. J Formos Med Assoc. 2005;104(3):157–63. 15818428

[31] Luo Z, Gardiner JC, Bradley CJ. Applying propensity score methods in medical research: pitfalls and prospects. Med Care Res Rev. 2010;67(5):528–54. 10.1177/1077558710361486 20442340 PMC3268514

[32] Miller J, Edwards LD, Agusti A, Bakke P, Calverley PM, Celli B, et al Comorbidity, systemic inflammation and outcomes in the ECLIPSE cohort. Respir Med. 2013;107(9):1376–84. 10.1016/j.rmed.2013.05.001 23791463

[33] Parappil A, Depczynski B, Collett P, Marks GB. Effect of comorbid diabetes on length of stay and risk of death in patients admitted with acute exacerbations of COPD. Respirology. 2010;15(6):918–22. 10.1111/j.1440-1843.2010.01781.x 20546185

[34] Gudmundsson G, Ulrik CS, Gislason T, Lindberg E, Brondum E, Bakke P, et al Long-term survival in patients hospitalized for chronic obstructive pulmonary disease: a prospective observational study in the Nordic countries. Int J Chron Obstruct Pulmon Dis. 2012;7:571–6. 10.2147/COPD.S34466 23055707 PMC3459657

[35] Wang Y, Stavem K, Dahl FA, Humerfelt S, Haugen T. Factors associated with a prolonged length of stay after acute exacerbation of chronic obstructive pulmonary disease (AECOPD). Int J Chron Obstruct Pulmon Dis. 2014;9:99–105. 10.2147/COPD.S51467 24477272 PMC3901775

[36] Litonjua AA, Lazarus R, Sparrow D, Demolles D, Weiss ST. Lung function in type 2 diabetes: the Normative Aging Study. Respir Med. 2005;99(12):1583–90. 10.1016/j.rmed.2005.03.023 16291079

[37] Lawlor DA, Ebrahim S, Smith GD. Associations of measures of lung function with insulin resistance and Type 2 diabetes: findings from the British Women's Heart and Health Study. Diabetologia. 2004;47(2):195–203. 10.1007/s00125-003-1310-6 14704837

[38] Forgiarini LA Jr., Kretzmann NA, Porawski M, Dias AS, Marroni NA. Experimental diabetes mellitus: oxidative stress and changes in lung structure. J Bras Pneumol. 2009;35(8):788–91. 19750332 10.1590/s1806-37132009000800011

[39] Esposito K, Nappo F, Marfella R, Giugliano G, Giugliano F, Ciotola M, et al Inflammatory cytokine concentrations are acutely increased by hyperglycemia in humans: role of oxidative stress. Circulation. 2002;106(16):2067–72. 12379575 10.1161/01.cir.0000034509.14906.ae

[40] Brennan AL, Gyi KM, Wood DM, Johnson J, Holliman R, Baines DL, et al Airway glucose concentrations and effect on growth of respiratory pathogens in cystic fibrosis. J Cyst Fibros. 2007;6(2):101–9. 10.1016/j.jcf.2006.03.009 16844431

[41] Marhoffer W, Stein M, Maeser E, Federlin K. Impairment of polymorphonuclear leukocyte function and metabolic control of diabetes. Diabetes Care. 1992;15(2):256–60. 1547682 10.2337/diacare.15.2.256

[42] Sode BF, Dahl M, Nordestgaard BG. Myocardial infarction and other co-morbidities in patients with chronic obstructive pulmonary disease: a Danish nationwide study of 7.4 million individuals. Eur Heart J. 2011;32(19):2365–75. 10.1093/eurheartj/ehr338 21875856

[43] Cazzola M, Bettoncelli G, Sessa E, Cricelli C, Biscione G. Prevalence of comorbidities in patients with chronic obstructive pulmonary disease. Respiration. 2010;80(2):112–9. 10.1159/000281880 20134148

[44] Park SK, Larson JL. The relationship between physical activity and metabolic syndrome in people with chronic obstructive pulmonary disease. J Cardiovasc Nurs. 2014;29(6):499–507. 10.1097/JCN.0000000000000096 24165700 PMC4032377

[45] Akpinar EE, Akpinar S, Ertek S, Sayin E, Gulhan M. Systemic inflammation and metabolic syndrome in stable COPD patients. Tuberk Toraks. 2012;60(3):230–7. 23030748

[46] Roglian P, Lucà G, Lauro D. Chronic obstructive pulmonary disease and diabetes. COPD Research and Practice. 2015;1:3.

[47] Glaser S, Kruger S, Merkel M, Bramlage P, Herth FJ. Chronic obstructive pulmonary disease and diabetes mellitus: a systematic review of the literature. Respiration. 2015;89(3):253–64. 10.1159/000369863 25677307

[48] Chatila WM, Thomashow BM, Minai OA, Criner GJ, Make BJ. Comorbidities in chronic obstructive pulmonary disease. Proc Am Thorac Soc. 2008;5(4):549–55. 10.1513/pats.200709-148ET 18453370 PMC2645334

[49] Agusti A, Calverley PM, Celli B, Coxson HO, Edwards LD, Lomas DA, et al Characterisation of COPD heterogeneity in the ECLIPSE cohort. Respir Res. 2010;11:122 10.1186/1465-9921-11-122 20831787 PMC2944278

[50] Bhatt SP, Dransfield MT. Chronic obstructive pulmonary disease and cardiovascular disease. Transl Res. 2013;162(4):237–51. 10.1016/j.trsl.2013.05.001 23727296

[51] Boudestein LC, Rutten FH, Cramer MJ, Lammers JW, Hoes AW. The impact of concurrent heart failure on prognosis in patients with chronic obstructive pulmonary disease. Eur J Heart Fail. 2009;11(12):1182–8. 10.1093/eurjhf/hfp148 19887495

[52] Macchia A, Rodriguez Moncalvo JJ, Kleinert M, Comignani PD, Gimeno G, Arakaki D, et al Unrecognised ventricular dysfunction in COPD. Eur Respir J. 2012;39(1):51–8. 10.1183/09031936.00044411 21700606

[53] Hawkins NM, Petrie MC, Jhund PS, Chalmers GW, Dunn FG, McMurray JJ. Heart failure and chronic obstructive pulmonary disease: diagnostic pitfalls and epidemiology. Eur J Heart Fail. 2009;11(2):130–9. 10.1093/eurjhf/hfn013 19168510 PMC2639415

[54] Lahousse L, Tiemeier H, Ikram MA, Brusselle GG. Chronic obstructive pulmonary disease and cerebrovascular disease: A comprehensive review. Respir Med. 2015;109(11):1371–80. 10.1016/j.rmed.2015.07.014 26342840

[55] Khedr EM, El Shinawy O, Khedr T, Abdel aziz ali Y, Awad EM. Assessment of corticodiaphragmatic pathway and pulmonary function in acute ischemic stroke patients. Eur J Neurol. 2000;7(5):509–16. 11054135 10.1046/j.1468-1331.2000.00104.x

[56] Martino R, Foley N, Bhogal S, Diamant N, Speechley M, Teasell R. Dysphagia after stroke: incidence, diagnosis, and pulmonary complications. Stroke. 2005;36(12):2756–63. 10.1161/01.STR.0000190056.76543.eb 16269630

[57] Yang YW, Chen YH, Wang KH, Wang CY, Lin HW. Risk of herpes zoster among patients with chronic obstructive pulmonary disease: a population-based study. CMAJ. 2011;183(5):E275–80. 10.1503/cmaj.101137 21343261 PMC3060212

[58] Lin HW, Chung CL, Lin YS, Yu CM, Lee CN, Bien MY. Inhaled Pharmacotherapy and Stroke Risk in Patients with Chronic Obstructive Pulmonary Disease: A Nationwide Population Based Study Using Two-Stage Approach. PLoS One. 2015;10(7):e0130102 10.1371/journal.pone.0130102 26158649 PMC4497597

[59] Cheng SL, Chan MC, Wang CC, Lin CH, Wang HC, Hsu JY, et al COPD in Taiwan: a National Epidemiology Survey. Int J Chron Obstruct Pulmon Dis. 2015;10:2459–67. 10.2147/COPD.S89672 26648708 PMC4648598

[60] Lin CW, Chen YY, Chen YJ, Liang CY, Lin MS, Chen W. Prevalence, risk factors, and health-related quality of life of osteoporosis in patients with COPD at a community hospital in Taiwan. Int J Chron Obstruct Pulmon Dis. 2015;10:1493–500. 10.2147/COPD.S85432 26251589 PMC4524376

[61] Niewoehner DE, Lokhnygina Y, Rice K, Kuschner WG, Sharafkhaneh A, Sarosi GA, et al Risk indexes for exacerbations and hospitalizations due to COPD. Chest. 2007;131(1):20–8. 10.1378/chest.06-1316 17218552

[62] Hurst JR, Vestbo J, Anzueto A, Locantore N, Mullerova H, Tal-Singer R, et al Susceptibility to exacerbation in chronic obstructive pulmonary disease. N Engl J Med. 2010;363(12):1128–38. 10.1056/NEJMoa0909883 20843247

