# Supplementary material for: Diabetes mellitus in patients with chronic obstructive pulmonary disease-The impact on mortality
Source: PLoS One. 2017 Apr 14;12(4):e0175794. doi: 10.1371/journal.pone.0175794 (PMC5391945; doi:10.1371/journal.pone.0175794)
Supplement: S1 Table — (DOCX) [file pone.0175794.s001.docx]

S1 Table. Definitions of comorbidities

| Comorbidities | ICD-9-CM codes |
| --- | --- |
| Hypertension | 401.x−405.x |
| Dyslipidemia | 270.0, 272.1 and 272.2 |
| Cerebrovascular disease | 430.x−438.x |
| Heart failure | 428.x, 584.x, 585.x and 586.x |
| Coronary artery disease | 410.x−414.x |
| Kidney disease | 582.x, 583−583.7, 584.x, 585.x, 586.x and 588.x |
| Liver disease | 571.2, 571.4−571.6, 572.2−572.8, 582−582.89 and 456−456.21 |
| Malignancy | 140.x−172.x, 174.x−195.8, 196.x−199.1, 200.x−208.x |

ICD-9-CM, International Classification of Diseases, Ninth Revision, Clinical Modification.
